# Physiological changes in vision during aging: perceptions of older adults and healthcare providers

**DOI:** 10.17533/udea.iee.v39n3e11

**Published:** 2021-11-08

**Authors:** Tattiana Dias de Carvalho Cordeiro, Luípa Michele Silva, Edilene Araujo Monteiro, Maria de Lourdes de Farias Pontes, Francine Golgheto Casemiro, Rosalina Aparecida Partezani Rodrigues

**Affiliations:** 1 Registered Nurse, MSc. Centro de Ciências da Saúde. Universidade Federal da Paraíba. João Pessoa-Paraíba- Brazil Email: tattianadccarvalho@gmail.com Universidade Federal da Paraíba Centro de Ciências da Saúde Universidade Federal da Paraíba João Pessoa Paraíba Brazil tattianadccarvalho@gmail.com; 2 Registered Nurse, Post doctorate. Assistant Professor Universidade Federal de Catalão- Goiás- Brazil Email: luipams@gmail.com Universidade Federal de Catalão Goiás Brazil luipams@gmail.com; 3 Registered Nurse, PhD. Assistant Professor Universidade Federal da Paraíba. João Pessoa-Paraíba- Brazil Email: edileneam06@gmail.com Universidade Federal da Paraíba Universidade Federal da Paraíba João Pessoa Paraíba Brazil edileneam06@gmail.com; 4 Registered Nurse, PhD. Assistant Professor Universidade Federal da Paraíba. João Pessoa-Paraíba- Brazil Email: profa.lourdespontes@gmail.com Universidade Federal da Paraíba Universidade Federal da Paraíba João Pessoa Paraíba Brazil profa.lourdespontes@gmail.com; 5 Gerontologist. PhD student. Escola de Enfermagem de Ribeirão Preto/USP. Ribeirão Preto- SP- Brazil. Email: francine_gc@hotmail.com Revista da Escola de Enfermagem da USP Escola de Enfermagem de Ribeirão Preto/USP Ribeirão Preto SP Brazil francine_gc@hotmail.com; 6 Registered Nurse, PhD. Full Professor, Escola de Enfermagem de Ribeirão Preto/USP. Ribeirão Preto- SP- Brazil. Email: rosalina@eerp.usp.br. Corresponding author Escola de Enfermagem de Ribeirão Preto/USP Ribeirão Preto SP Brazil rosalina@eerp.usp.br

**Keywords:** geriatric nursing, aged, vision, ocular, aging., enfermería geriátrica, anciano, visión ocular, envejecimiento., enfermagem, geriátrica, idoso, visão ocular, envelhecimento.

## Abstract

**Objective:**

To identify the physiological changes in older adults’ vision during the aging process.

**Methods:**

An exploratory, descriptive study with a qualitative approach was conducted with 20 older adults and six healthcare providers who worked with older adults in João Pessoa, Paraíba (Brazil). The Focus Group Technique was used for data collection, with the collected information subsequently being submitted to Inductive Thematic Analysis using textual analysis software.

**Results:**

The physiological changes related to vision were described by both the older adults and healthcare providers using the following words: vision, difficulty; see; cataract; glasses; surgery; more; age; eye; and no. These terms represent declines in vision resulting from advancing age which significantly modify the daily lives of older adults and their families.

**Conclusion:**

The perception of the older adults and the healthcare providers who care for them regarding the physiological changes in vision throughout the aging process shows that the loss of visual acuity significantly affects the daily life of older adults and their families.

## Resumen

**Objetivo:**

Describir la percepción de las personas mayores y de los profesionales que trabajan con ellas sobre los cambios fisiológicos en la visión durante el proceso de envejecimiento.

**Métodos:**

Estudio exploratorio, descriptivo con análisis cualitativo, realizado con 20 adultos mayores y seis profesionales de la salud que trabajan con adultos mayores en Paraíba (Brasil). Para la recolección de los datos se utilizó la técnica de grupo focal. A la información recopilada se le realizó el análisis de temática inductiva con la ayuda del software de análisis textual.

**Resultados:**

Tanto los adultos mayores como los profesionales describieron los cambios fisiológicos relacionados con la visión con las siguientes palabras: visión, dificultad, ver, catarata, anteojos, cirugía, más, edad, ojo y “no”.

**Conclusión:**

La percepción de los adultos mayores y de profesionales que los atienden sobre los cambios fisiológicos en la visión muestra que la pérdida de la agudeza visual modifica significativamente la vida cotidiana del anciano y la de su familia.

## Resumo

**Objetivo:**

Descriver a percepção de idosos e profissionais que trabalham com idosos sobre as alterações fisiológicas da visão durante o processo de envelhecimento.

**Métodos:**

Estudo exploratório, descritivo, com análise qualitativa, realizado com 20 idosos e seis profissionais de saúde que trabalham com idosos em João Pessoa, Paraíba (Brasil). Para a coleta de dados, utilizou-se a Técnica de Grupos Focais, sendo que os dados foram submetidos à Análise Temática Indutiva com auxílio de um software de análise textual.

**Resultados:**

As alterações fisiológicas relacionadas à visão foram descritas tanto pelos idosos quanto pelos profissionais por meio das seguintes palavras: visão, dificuldade; enxergar; catarata; óculos; cirurgia; mais; idade; olho e não. Esses termos representam declínios da visão decorrentes do avanço da idade que modificam significativamente o cotidiano dos idosos e seus familiares.

**Conclusão:**

A percepção dos idosos e dos profissionais que os cuidam sobre as alterações fisiológicas da visão mostra que a perda da acuidade visual modifica significativamente o cotidiano do idoso e de sua família.

## Introduction

Although not all older adults have health problems, the aging process is marked by physiological impairments that often generate direct impacts on the health and social security systems. Increasingly, technological advances in health are providing an extension of life, with gains in quality accompanied by a reduction in mortality rates from non-communicable diseases and greater access to better public and private health services.(1) In older adults with physiological impairments, attention should be paid to the possibility that the sense organs are being impacted. Sensory deficits occur gradually, imposing restrictions to varying degrees on the older adult’s daily activities, as well as affecting the spheres of safety and independence, general well-being and quality of life, with it being important to assess whether or not they are symptomatic of an underlying disease.(2)

The eye is the organ of the sensory system that undergoes the most impactful changes. In general, there are some impairment in the structure of the eye, in the visual function, from the fourth decade of life, for several reasons: loss of elasticity of the eyelids, making them soft and wrinkled, intrinsic eye diseases (cataracts) and acquired neurological or systemic diseases (diabetes), for example.(3) In addition to the examples mentioned, other physiological changes in vision in older adults should be highlighted, such as: decreased visual acuity, narrowing of the visual field, sensitivity to bright light, poor night vision, confused dark colors and dry eyes.(4)

Accordingly, healthcare providers, especially nurses, have the role of ensuring the necessary care to promote well-being and quality of life for older adults, maintaining their autonomy and independence.(3) In the multidimensional assessment of the older adult, the vision is the sense that establishes the relationship of the person with others and with the environment, therefore, in nursing care it is essential to highlight this knowledge in the area, which is one of the gaps in nursing. It is considered essential to contribute to the formation of healthcare providers so that they are better prepared to comprehend the aging process and the care demands at this stage of life, supported by scientific research. For this purpose, the following research questions were established: What is the perception of older adults regarding the physiological changes in their vision during aging? What is the perception of healthcare providers who work with older adults regarding the difficulties related to changes in the vision of these people assisted during the aging process? The aim of the study was to analyze the perception of older adults and healthcare providers who work with older adults regarding the physiological changes in the older person’s vision throughout the aging process.

## Methods

This was an exploratory, descriptive and qualitative study. Data were collected using the Focus Group (FG) technique and analyzed according to Inductive Thematic Analysis. The field research took place in two care spaces for older adults: at the *Centro de Atenção Integral à Saúde do Idoso* (CAISI) in the city of João Pessoa/PB and at the *Instituto Paraibano de Envelhecimento* of the Federal University of Paraíba-UFPB (IPE/UFPB). The CAISI is a service of the Municipal Health Department of João Pessoa that provides specialized medical care and assistance in the areas of physiotherapy, speech therapy, psychology, nutrition, and nursing, as well as performing operative groups, with activities aimed at prevention and health promotion.^5^

The study population consisted of older adults who attended the operative groups of the CAISI and healthcare providers from the Graduate Program in Gerontology (students and professors) of the aforementioned public university, which has a multidisciplinary nature. It should be highlighted that the use of these two varied scenarios favored the apprehension of different knowledge and, therefore, the understanding of the subject. The inclusion criteria for the older adults were: being 60 years of age or over; availability to be present at more than one meeting; and presenting good communication and sufficient cognitive status to answer the questions, verified through simple questions such as name, date of birth and day of the month, week and current year (2019). The inclusion criteria for the healthcare providers were: to have academic training and/or to exercise practical activity in the care of older adults; and to be available to be present at more than one meeting. One of the researchers visited three operative groups at the CAISI in order to present the research proposal and invite the older adults. After the invitation, the older adults that were interested provided data such as name, age and telephone numbers in order to inform them about the dates and times of the groups. A total of 20 older adults agreed to participate in the study. For the selection of the healthcare providers, one of the researchers contacted them in advance by telephone to explain the research and perform the invitation, which was sent by e-mail. Of the 20 healthcare providers invited, only six participated in the study, due to the lack of spare time.

To carry out the study, two focus groups were formed in order to develop the theme, since differences and/or similarities can interfere in the analysis of information. For the intentional sample, the older adults’ own experiences in relation to vision changes and the experiences and knowledge of the gerontology professionals about the subject were considered. Accordingly, for this study, two Focus Groups were formed: the 1st with the older adults (FGO) and the 2nd with the healthcare providers (FGP). A meeting was also held with the participants of the focus groups (FGO and FGP), to validate the final result of the two FGs. The focus groups and the meeting were held in a room at the *Instituto Paraibano de Envelhecimento* at the Federal University of Paraíba-UFPB, after the participants consented to participate, on a previously scheduled date and time.

Data collection took place in May 2019, with only one meeting per focus group. Participants totaled 26, 20 in the FGO and six in the FGP, all those who were invited and agreed to participate in the study attended on the scheduled date and time. Each meeting lasted two hours. For the performance of the groups, there was a moderator (Master’s degree holder and nurse), with the function of organizing the meeting and encouraging the discussion process; an observer, to synthesize and record the group discussions; and collaborators (one graduate student in nursing, one graduate student in gerontology and two Nursing Doctorate holds). The sessions were audio-recorded, with subsequent transcription and data analysis.

In the FGO, an interview was previously carried out with the participants to characterize the older adults, considering the following variables: age (in years), sex (female, male), marital status (single, married, separated/divorced, widowed), education (illiterate, can read and write, fundamental education, high school education, higher education), difficulty in one of the five senses, with emphasis on vision and the use of glasses or other orthosis. To conduct the focus group, the following guiding questions were established: Do you have any changes or difficulties in your sense of vision? Which one or which ones? For how long? Have you been to the doctor because of this difficulty? What is the perception in relation to this change or difficulty? Do you know any older adult who has altered vision?

In the FGP, the meeting with the providers followed the same steps as the FGO. To characterize the participants, a questionnaire was used containing the following variables: age (in years), gender (female, male), professional training, time since graduation and occupation. There was a second questionnaire, with eight questions, to identify the experiences of these professionals in caring for older adults with vision impairment. To conduct the focus group, the following guiding questions were used: Have you ever attended an older adult with sensory-visual alteration? What was the difficulty presented by them? How old were they? How long had they had this alteration? Did you find it difficult to provide this older adult with care? What difficulty? Had this older adult already sought professional care to treat this difficulty? Do you know the diagnosis?

The purpose the third meeting was to present the results of the alterations reported by the older adults and the healthcare providers’ experiences in a single panel for debate and validation of what had been presented during the sessions and final analysis. All dialogues produced during the focus groups were fully transcribed for data organization and further analysis. Sociodemographic data were entered into an electronic spreadsheet in the Microsoft Excel program. Descriptive statistics and the Inductive Thematic Analysis technique(6) were performed, using the *R pour les Analyses multidimensionnelles de textes et de questionnaires* (IRaMuTeQ) software.(7) The texts produced in the FGs were analyzed using IRaMuTeQ. In the software, the chosen analyses were: 1) Word cloud - formed from the frequency distribution of words and obtained through a lexical analysis simpler than similarity analysis(6); 2) Similarity analysis - which is based on graph theory, enabling the identification of co-occurrences between the words and the connection between them, as well as the visualization of the representational structure organized through this type of analysis.(7)

All 20 older adults and the six providers signed a consent form, in duplicate, a copy of which was retained by the study participant. This study met all the ethical requirements established in Resolution 466/2012, of the National Health Council, having been approved by the Research Ethics Committee of UFPB (Authorization No. 2.190.153 and CAAE 67103917.6.0000.5188). The research at the CAISI was authorized by the Health Department of João Pessoa and by the Health Education Management.

## Results

### Characterization of the Focus Group participants

The FGO consisted of 20 older adults, between 70 and 91 years of age; most female. Of these, 19 reported using eyeglasses, more than half (65%) had only completed primary education and half (10) were widowed. In the FGP, participants were six healthcare providers who were directly linked to care activities for older adults, aged between 29 and 52 years, four being female and two male, with the time since graduation ranging between one and 28 years. Among the providers, there were three nurses, a physical educator, a Management and Art technician from the Municipal Health Department and an Older Adults Club and the coordinator of the Strategic and Participatory Management Support Center of the Municipal Health Department, both responsible for the operative groups of the CAISI. In common, all the providers were studying for master’s degrees in the Professional Master’s Degree Program in Gerontology at the Federal University of Paraíba.

### Focus Group Analysis

In the analysis of IRaMuTeQ, in the FGO, 36 text segments were formed, with a total of 1,272 occurrences, of which 364 words were analyzable. A total of 205 different words were identified with a single occurrence, which corresponds to 56.32% of the total analyzed and 16.12% of the occurrences. In the FGP, 38 text segments were formed, with a total of 1,347 occurrences, of which 642 words were analyzable. A total of 270 different words were identified with a single occurrence, which corresponds to 58.44% of the total analyzed and 20.04% of the occurrences.

**Figure 1:**
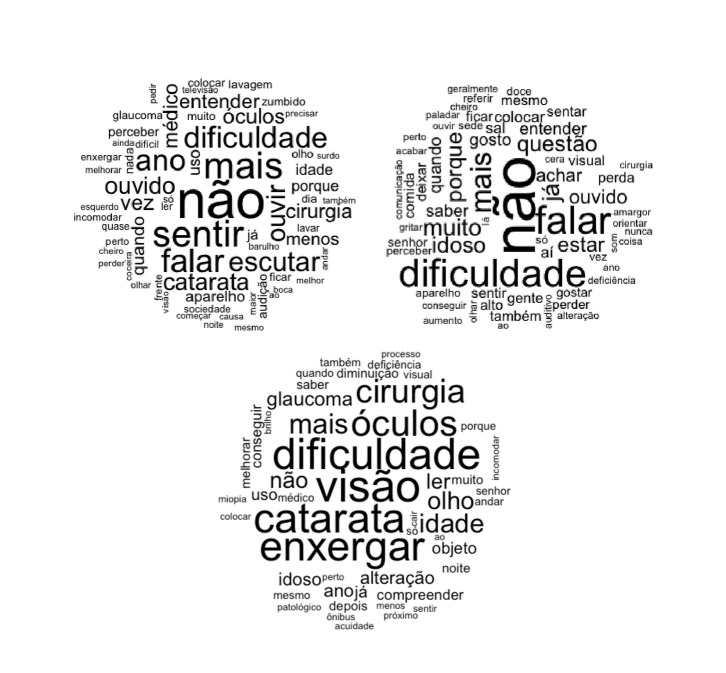
Word clouds formed from the focus groups, the left cloud for the older adult group, the right cloud for the healthcare provider group and below the cloud for the third meeting, João Pessoa, Paraíba, Brazil, 2019

In the word cloud, on the left, in Figure 1, the ones with greater visibility and their respective frequencies were: no (*f*=39); feel (*f*=26); more (*f*=26); speak (*f*=26); difficulty (*f*=20); year (*f*=20); listen (*f*=18); listen (*f*=17); ear (*f*=15); cataract (*f*=15); glasses (*f*=13); time (*f*=13); doctor (*f*=12; surgery (*f*=12) and understand (*f*=12). In the right cloud in Figure 1, the words with greater visibility and frequency were: no (*f*=39); speak (*f*=24); difficulty (*f*=24); more (*f*=17); a lot (*f*=13); already (*f*=13); question (*f*=12); because (*f*=12); elderly (*f*=12); be (*f*=11) and ear (*f*=10).In the center word cloud, below the two clouds, which is a combination of what was reported in the third meeting, the words with greater visibility and respective frequency were: vision (*f*=24); difficulty (*f*=23); see (*f*=22); cataracts (*f*=22); glasses (*f*=19); surgery (*f*=17); more (*f*=14); age (*f*=13); eye (*f*=12) and no (*f*=11).

Left: colcar = put; lavagem = wash; televisão = television; pedir = ask; entender = understand; zumbido = tinnitus; muito = a lot; óculos = glasses; precisar need; glaucoma = glaucoma; perceber = perceive; ainda = still; dificil = difficult; enxergar = see; melhorar = improve;nada = nothing; só = only; esquerdo = left; incomodar = trouble; quase = almost; perto = near; cheiro = smell; perder = lose; coceira = itch; olhar = look; frente = front; visão = vision; aparelho = device; sociedade = society; começar = start; causa = cause; noite = night; mesmo = same; maior = bigger; audição = hearing; ao = to; boca = mouth; ficar = stay; melhor = best; andar = walk; barulho = noise; menos = less; lavar = wash; cirurgia = surgery; dia = day; porque = why; idade = age; surdo = deaf; olho = eye; dificuldade = difficulty; não = no; sentir = feel; mais = more; falar = speak; ano = year; escutar = listen; ouvir = hear; ouvido = ear; catarata = cataract; médico = doctor; uso = use; vez = turn; quando = when;

Right: dificuldade = difficulty; geralmente = generally; doce = sweet; referir = refer; mesmo = same; cheiro = smell; sentar = sit; paladar = taste; ficar = stay; colocar = put; ouvir = hear; sede = thirsty; sal = salt; entender = understand; gosto = taste; perto = close; questão = question; acabar = finish; cera = wax; visual = visual; cirurgia = surgery; perda = loss; ouvido = ear; saber = know; gritar = scream; muito = a lot; falar = speak; amargor = bitterness; orientar = guide; nunca = never; coisa = thing; só = only; aí = there; estar = be; vez = turn; ano = year; deficiencia = deficiency; gostar = like; perder = lose; alteração = alteration; gente = people; sentir = feel; alto = loud, também = also; ao = to; aumento = increase; conseguir = manage; aparelho = device; perceber = perceive; senhor = Mr; idoso = older adult; comunicação = communication; comida = food; deixar = leave; quando = when; porque = why; mais = more; não = no; já = already; som = sound;

Center: processo = process; também = also; deficiencia = deficiency; quando = when; diminuaçao = decrease; visual = visual; saber = know; glaucoma = glaucoma; cirurgia = surgery; mais = more; óculos = glasses; porque = why; dificuldade = difficulty; não = no; visão = vision; ler = read; muito = a lot; senhor = Mr; andar = walk; olho = eye; uso = use; medico = doctor; miopia = myopia; colocar = put: catarata = cataract; idade = age; só = only; ao = to; enxergar = see; objeto = object; noite = night; idoso = older adult; perto = close; alteraçao = alteration; mesmo = same; ano = year; já = already; compreender = understand; patológico = pathological; depois = after; menos = less; sentir = feel; ónibus = bus; proximo = next; acuidade = acuity; melhor = better; conseguir = manage; brilho = bright; cair = fall; incomodar = trouble

**Figure 2:**
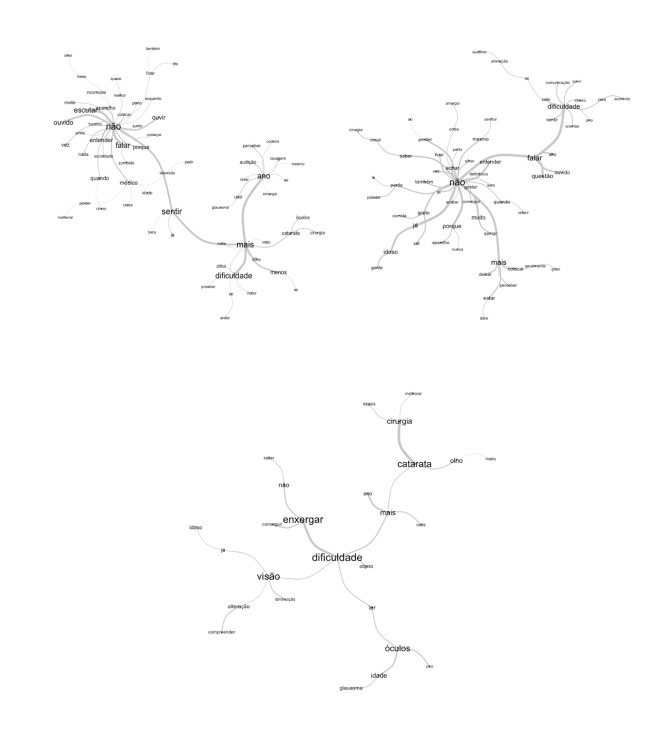
Maximum trees formed from the focus groups, the left tree for the older adult group, the right tree for the healthcare provider group and below the tree for the third meeting, João Pessoa, Paraíba, Brazil, 2019.

In the maximum tree, Figure 2 on the left, generated from the older adult group, the formation of several nodes (nuclei) stands out, with the words “no”, “feel”, “more”, “difficulty” and “year”; the nodes are linked to other peripheral elements that are related to the difficulties that the older adults had related to vision and to coping with their problems with advancing age. The second tree on the right, which emerged from the providers’ statements, has nodes represented by the words “no”, “speak”, “difficulty” and “more”, in the providers’ perception, age is permeated by difficulties and losses, with communication being an important element in the comprehension of the problems reported by the older adults. All the elements listed by the tree represent not only the decline in vision due to age, but other senses that may be altered, such as hearing. In the third tree there is the formation of two nodes (nuclei), the first between the words “see” and “difficulty”, this node is linked to other peripheral elements that are related to difficulty in seeing, reading and wearing glasses with advancing age. The second node, represented by the words “cataract” and “surgery”, was linked to the first by the word “more”, which is directly linked to the word “difficulty”. All the elements listed by the tree represent the declines in vision due to age.

Left: não = no; também = also; dia = day; ficar = stay; olhar = look; frente = front; incomodar = trouble; quase = almost; melhor = best; perto = near; colcar = put; aparelho = device; muito = a lot; escutar = listen; surdo = deaf; ouvir = hear; ouvido = ear; barulho = noise; ainda = still; vez = turn; nada = nothing; entender = understand; sociedade = society; quando = when; médico = doctor; zumbido = tinnitus; falar = speak; porque = why; cheiro = smell; perder = lose; melhorar = improve; causa = cause; idade = age; televisão = television; pedir = ask; esquerdo = left; sentir = feel; boca = mouth; já = already; ano = year; coceira = itch; perceber = perceive; lavagem = wash; mesmo = same; audição = hearing; lavar = wash; uso = use; glaucoma = glaucoma; enxergar = see; ao = to; mais = more; olho = eye; noite = night; visão = vision; menos = less; só = only; óculos = glasses; catarata = cataract; cirurgia = surgery; dificuldade = difficulty; dificil = difficult; ler = read; maior = bigger; andar = walk; precisar = need;

Right: dificuldade = difficulty; sede = thirsty; só = only; alteração = alteration; auditivo = auditory; comunicação = communication; ouvir = hear; sentir = feel; orientar = guide; ano = year; cera = wax; aumento = increase; cheiro = smell; falar = speak; alto = loud; questão = question; ouvido = ear; não = no; olhar = look; senhor = Mr; mesmo = same; perto = close; amargor = bitterness; coisa = thing; ao = to; perder = lose; achar = find; ficar = stay; vez = turn; saber = know; visual = visual; cirurgia = surgery; também = also; perda = loss; paladar = taste; lá = there; comida = food; gosto = taste; aí = there; já = already idoso = older adult; gente = people; sal = salt; porque = why; aparelho = device; nunca = never; sentar = sit; muito = a lot; conseguir = manage; referir = refer; quando = when; gostar = like; entender = understand; deficiencia = deficiency; som = sound; acabar = finish; mais = more; deixar = leave; colocar = put; geralmente = generally; gritar = scream; perceber = perceive; estar = be; doce = sweet

Center: dificuldade = difficulty; objeto = object; óculos = glasses; ler = read; uso = use; idade = age; glaucoma = glaucoma; visão = vision; diminuaçao = decrease; alteraçao = alteration; compreender = understand; já = already; idoso = older adult; enxergar = see; conseguir = manage; não = no; saber = know; mais = more; ano = year; noite = night; catarata = cataract; olho = eye; muito = a lot; cirurgia = surgery; melhor = better; depois = after

## Discussion

From the perceptions of the older adults and healthcare providers who worked with older adults, it was possible to visualize how changes related to vision are reported and perceived by the different groups. However, when viewing the results, many common points permeated the reports. In view of the main physiological changes in vision perceived by the older adults and described by the providers during the care for older adults, the most frequently mentioned words by the participants were evidenced: vision, difficulty; see; cataract; glasses; surgery; more; age; eye and no. This result corroborates the findings of the World Vision Report, according to which visual impairment is observed in 80% of cases from 50 years of age onwards.(8) The participants’ statements denote that the onset of diseases related to vision such as cataracts, retina, glaucoma, myopia, correction and degeneration are common in aging. Researchers from Nepal concluded that, with population aging, retinal diseases are the main cause of blindness.(9) It is important to highlight that the older adults experience the problem and report it frequently.

The older adults, in addition to reporting difficulties related to vision, also reported declines in other senses. In the literature, the difficulty in seeing nearby objects is reported as being common among older adults, which makes it difficult, for example, to read.(10) This change is due to aging, such as age-related macular degeneration, which still has no possible treatment and has been receiving more attention recently due to the high prevalence figures and the negative effect it imposes on the quality of life.(8) Access to the use of eyeglasses and surgery is an important factor in people’s quality of life, as these allow for the necessary correction, as well as the reduction of the alarming numbers of people with visual impairment and blindness.(11) The main causes of visual impairment and blindness are the reversible changes that greatly affect the older population and that are treatable with surgery, glasses or contact lenses, mainly cataracts and refractive errors.(12,13)

Other words indicated that the providers’ concern should not only be with identifying the problem and concluding a diagnosis, with it also being necessary to consider the complications of visual decline as alterations in the quality of life and social aspects.(14) In the focus group cloud words such as bus, fall, night, glare, feel and walk, related to daily difficulties experienced by the older adults as a result of impaired vision, were also identified. In the literature, these words are cited by older adults with senile cataract, who need a greater amount of light to see better, especially at night, in addition to denoting decreased acuity, blurring, distortion, and loss of brightness and color.(15)

Older adults, faced with the difficulty of seeing or even the lack of vision, may present depression and anxiety, limited mobility, cognitive decline, dementia, and a greater risk of falls, fractures and mortality, as well as other consequences such as institutionalization.(16-22) In Germany, researchers warned of the need to promote eye care for older adults. The authors found, in addition to ocular disorders increasing with age, severe visual impairment and blindness in 136 older adults out of 600 respondents, particularly due to age-related pathologies, such as age-related macular degeneration, cataracts and glaucoma.(23)

In addition to the difficulties involved in the participation of the older adults in activities outside their homes, other themes arising from words such as “analysis”, “evaluation”, “vision”, “examination”, “doctor” and “evaluation” should be focused on, as these are part of the routines of health services. The presence of these words is in line with some findings in the literature, such as those reported in a study carried out in Fortaleza with 172 older adults treated at an Urgency and Emergency Hospital. According to the study, 28.5% of the participants had poor eyesight, with men having the most complaints regarding vision decline.(24)

In the UK, researchers conducted a hospital survey on admissions of people with a secondary diagnosis of glaucoma and hospitalization for falls with or without glaucoma as a secondary diagnosis. The survey considered the period of six years and showed that for every eight falls in older adults, one had glaucoma as an important factor, leading to the need for hospitalization and generating a high personal and financial cost, estimated in the study at around £1.2 million during the period investigated. The authors suggested that there is a greater probability of hospitalization due to falls for patients with glaucoma than among those not affected by this disease (prevalence of 0.85% versus 0.16%.(25)

In the maximum tree, the formation of nodes (nuclei) was identified, with the most significant of them being between the words “visual” and “disability”. A secondary node, which should also be noticed, is formed by the words “vision” and “difficulty”, which connects to other peripheral elements that emphasize not being able to see and advancing age as factors of visual impairment in seniors. The causes, that is, visual alterations, the object of this study, impose important consequences on the lives of the older adults and their families, as well as on society.(10) In all the analyses, the terms show a connection between “disability” and “visual” and between “difficulty” and “vision”. These findings are in accordance with the International Classification of Functioning, Disability and Health (ICF), according to which the use of the term “difficulty” as a qualifier to measure visual function contributes to a better comprehension of the visual impairment-health status.(25)

Linked to the main node, elements related to impaired vision were identified, which can reach the most severe degree, blindness. Accordingly, the assessment should be carried out early, to identify the type of change and, consequently, the appropriate treatment, in order to correct errors or postpone any more serious involvement due to lack of necessary care for changes such as glaucoma, myopia and cataract.(16) Restricted access to eye care, especially in low- and middle-income countries, associated with global aging and changes in people’s lifestyles, has significantly contributed to the increase in the number of individuals with visual impairments, according to estimates, this problem is 5% in developed countries and reaches 50% in the poorest regions of the world.(8) This is an important fact, since visual impairment causes negative consequences in people’s lives, affecting their routine and quality of life.(20) Regarding these consequences, there are, in the maximum tree, elements that may reflect the concern of providers who care for older adults to identify the main changes related to vision and the consequences of these losses. It is also possible to visualize terms that indicate the reduction of vision as a limiting factor in the life of the older adults, due to the difficulty in seeing objects and reading, characterizing changes in their daily activities.(17)

The results are shown in word clouds, in which it is possible to identify the perception of changes, cited by the older adults such as “trouble”, “feel”, “understand”, “read”, “object”, “night”, “brightness”, “difficulty”, “fall”, “bus”, “institutionalize”, “social”, “life”, “quality” and “support” in reference to the individual, family and social implications that change the daily life of this population.

The perceptions of the older adults regarding visual alterations described in the focus groups were related to advancing age and are characterized as the main causes of visual impairment and blindness worldwide. According to the literature, changes that are common in the older adults include: cataracts, refractive errors, especially myopia and presbyopia, age-related macular degeneration, glaucoma, and diabetic retinopathy.(18,19) It should be highlighted that many of these changes appear in all the analyses of this study.

It should also be noted that the tree shows a relationship between several terms that denote that there is an intersection between difficulty in vision, aging, visual changes and compromised quality of life. These factors constitute a warning for Brazilian researchers, since the country is not prepared to deal with the needs of aging alterations that can generate this clinical condition (visual problems), requiring a continuous and multidisciplinary organization of the health system.^1^ A study carried out in the capital of Paraíba with 34 nursing care providers from a university hospital showed that they had difficulties in communicating with visually impaired clients and that this could compromise the care. The researchers reinforced the need for the education organizations to prepare future providers, as well as the encouragement of training by the service providers.(10)

Two limitations of this study can be highlighted: the reduced participation of healthcare providers who work with older adults, although our focus was not on generalizing the results, and the limited number of publications on the physiological visual alterations that affect Brazilian older adults, with no censuses or population studies that show these changes. In our view, the information in this regard is mainly concentrated in textbooks, which makes the development of research in the area urgent. Therefore, the development of research in the field of nursing in ophthalmology is urgently needed. In summary, it is noticeable in the word clouds and in the maximum tree that vision problems are directly related to advancing age and, as a consequence, affect the quality of life of older adults. It should be emphasized that the results obtained in the focus groups, through the statements of both the older adults and the healthcare providers, show how the difficulty of seeing and the problems related to vision directly affect the life of older adults and their activities of daily living, mainly the social aspects.

For older adults, visual difficulties generate impacts both at an individual and collective level, resulting in psychological, social and economic problems, which will directly imply loss of self-esteem, status, occupational restrictions and decrease in income. It is also important to highlight that these situations end up generating dependence, communication problems and loss of autonomy for the older adults, who will depend on others for their routine daily care.(17) Accordingly, this study contributes not only to the work of the providers who care for older adults, but reiterates the need for nursing care providers to be aware of changes in older adults’ visual acuity inherent to aging and that can affect the daily lives of these people, with a significant impact on their quality of life. The perception of the older adults and healthcare providers regarding the physiological changes in the older adults’ vision throughout the aging process shows how the older adults’ vision in this process impacts on their daily lives.

Measures for the implementation of preventive actions and monitoring of older adults regarding visual acuity must be included in the multidimensional assessment in health services, and this information must be recorded and monitored by a multidisciplinary team. Older adults, with their visual alterations are objects of study, however interventions must be implemented to offer them quality of life, even with visual alterations.
